# Effect of 3 and 6 mg/kg of caffeine on fat oxidation during exercise in healthy active females

**DOI:** 10.5114/biolsport.2023.121321

**Published:** 2022-11-18

**Authors:** David Varillas-Delgado, Millán Aguilar-Navarro, Alejandro Muñoz, Álvaro López-Samanés, Carlos Ruiz-Moreno, María Posada-Ayala, Francisco J. Amaro-Gahete, Juan Del Coso, Jorge Gutiérrez-Hellín

**Affiliations:** 1 Universidad Francisco de Vitoria, Faculty of Health Sciences, Madrid, Spain; 2 Camilo José Cela University, Exercise Physiology Laboratory, Madrid, Spain; 3 Universidad Francisco de Vitoria, Faculty of Experimental Sciences, Madrid, Spain; 4 Department of Medical Physiology, Faculty of Medicine, University of Granada, Granada, Spain; 5 Centre for Sport Studies, Rey Juan Carlos University, Madrid, Spain

**Keywords:** Stimulant, Phytochemical, Substrate oxidation, Endurance exercise, Female athletes

## Abstract

The aim of this study was to investigate the effect of 3 and 6 mg of caffeine per kg of body mass (mg/kg) on whole-body substrate oxidation during an incremental cycling exercise test in healthy active women. Using a double-blind placebo-controlled counterbalanced experimental design, 14 subjects performed three identical exercise trials after the ingestion of 3 or 6 mg/kg of caffeine or placebo. The exercise trials consisted of an incremental test on a cycle ergometer with 3-min stages at workloads from 30 to 70% of maximal oxygen uptake (VO_2_max). Substrate oxidation rates were measured by indirect calorimetry. During exercise, there was a significant effect of substance (F = 5.221; p = 0.016) on fat oxidation rate. In comparison to the placebo, 3 mg/kg of caffeine increased fat oxidation rates at 30 to 60% of VO_2_max (all p < 0.050) and 6 mg/kg at 30 to 50% of VO_2_max (all p < 0.050). There was also a significant effect of substance (F = 5.221; p = 0.016) on carbohydrate oxidation rate (F = 9.632; p < 0.001). In comparison to placebo, both caffeine doses decreased carbohydrate oxidation rates at 40 to 60% VO_2_max (all p < 0.050). The maximal rate of fat oxidation with placebo was 0.24 ± 0.03 g/min, which increased with 3 mg/kg to 0.29 ± 0.04 g/min (p = 0.032) and to 0.29 ± 0.03 with 6 mg/kg of caffeine (p = 0.042). Acute intake of caffeine improves the utilization of fat as a fuel during submaximal aerobic exercise in healthy active women with an effect of similar magnitude after the intake of 3 and 6 mg of caffeine per kg of body mass. Thus, the use of 3 mg/kg of caffeine would be more recommended than 6 mg/kg for women seeking increased fat utilization during submaximal exercise.

## INTRODUCTION

Caffeine is one of the most widely used phytochemicals as an ergogenic aid [1], with strong evidence supporting its positive effects on sport performance [2–4]. Today there is a consensus to consider acute caffeine intake as an ergogenic supplementation protocol when caffeine is ingested in a dose of 3 to 6 mg per kg of body mass [2]. Beyond the performance benefits in the sport context, acute caffeine intake can produce other physiological changes during exercise, such as increased fat oxidation. The effect of caffeine to shift substrate oxidation towards a higher reliance on fat is present during submaximal aerobic exercise [5] and when ingested in doses ranging from 3 to 6 mg/kg [6–10]. Recently, it was found that the effect of caffeine to enhance fat oxidation during submaximal aerobic exercise is of similar magnitude with 3 and 6 mg of caffeine per kg of body mass [11]. However, was the case in the investigation of the ergogenic effect of caffeine [12], most of the findings regarding the potential effect of caffeine to augment fat utilization during aerobic exercise have been obtained in samples of male participants or mixed samples where women constituted only a small part of the sample. Hence, from the current literature on the topic, it is not possible to determine with confidence whether women benefit from acute caffeine intake to enhance fat oxidation during exercise and whether this effect is obtained in the range of 3 to 6 mg/kg as occurs in men.

To date, there are only three investigations indicating that, in women, a dose of ~6 mg/kg produces larger performance improvements than lower doses of ~3 mg/kg of caffeine or less [13–15]. Against this background, it is possible that women obtain benefits from fat oxidation during exercise of higher magnitude with 6 than with 3 mg/kg of caffeine. Therefore, the aim of this study was to investigate the effect of 3 and 6 mg of caffeine per kg of body mass (mg/kg) on whole-body substrate oxidation during an incremental cycling exercise test in healthy active women. We hypothesized that fat oxidation rates would be higher with both doses of caffeine over a placebo, but the magnitude of the effect would be higher with 6 mg/kg than with 3 mg/kg of caffeine.

## MATERIALS AND METHODS

### Participants

Fourteen healthy active women volunteered to participate in this study (Table 1). Participants were considered active because all of them performed at least 60 min of exercise per day for at least 4 days per week for the two years prior to the investigation. Participants practised different aerobic sport disciplines such as road cycling, mountain biking, endurance running and triathlon. An a priori sample size calculation indicated that 14 participants were needed to obtain statistically significant differences in the maximal fat oxidation rate with caffeine with respect to the placebo. This a priori sample size was calculated to obtain an effect size of 1.01 in Cohen’s d units (statistical power of 80% with type I error set at 5%), based on a previous investigation that obtained this effect when using the same dose of caffeine during an incremental exercise test [7]. The required sample size was determined using G*Power software [16]. All participants were non-smokers, had no history of cardiopulmonary diseases, suffered no musculoskeletal injuries in the previous 6 months, had a regular duration of their menstrual cycle during the previous 6 months and had no menstrual disorders such as dysmenorrhoea, amenorrhoea or strong symptoms associated with premenstrual syndrome. Participants were low caffeine consumers (i.e., < 50 mg caffeine per day in the previous 2 months) as defined by Filip et al. [17] and measured by a modified version of the validated questionnaire by Bühler et al. [18]. All participants signed a written informed consent form to participate in the research. The experimental procedure of this study was in accordance with the latest version of the Declaration of Helsinki and was approved by the local University Ethics Committee (IRB number UFV 18/2020).

**TABLE 1:** Participants’ age, morphological characteristics, and maximal values at the end of a maximal ramp test on a cycle ergometer.

Variable (units)	Mean ± SD	Range
Age (yr)	26.0 ± 6.0	19–39
Body mass (kg)	60.7 ± 7.9	47.9–73.0
Height (m)	167 ± 6	156–175
Fat mass (%)	14.9 ± 3.6	8.02–30.5
VO_2_max (ml/kg/min)	40.3 ± 5.4	32.9–50.2
Maximal heart rate (beats/min)	180 ± 7	170–197
Maximal respiratory quotient	1.22 ± 0.06	1.13–1.37
Maximal Borg’s scale rating (a.u.)	20.0 ± 0.2	19–20

Arbitrary units (a.u.). Data are presented as mean ± standard deviations (SD) with minimal and maximal values (range).

### Experimental design

A randomised, counterbalanced, double-blind, placebo-controlled design was used in this experimental investigation. Each participant completed 3 identical experimental trials after conducting a pre-experimental maximal oxygen consumption test. All experimental trials were separated by at least 3 days to allow for full recovery and caffeine washout. In a randomised, counterbalanced order, the participants ingested either (i) 3 mg/kg of caffeine (100% purity; CAF, Bulk Powders, UK), (ii) 6 mg/kg of caffeine or (iii) a placebo (cellulose; 100% purity, Guinama, Spain). The substances were administered in an opaque, unidentifiable capsule and ingested with 150 mL of water 60 minutes before the beginning of the experimental trials while a researcher visually verified the ingestion. An alphanumeric code was assigned to all trials and the randomization of trial order was performed by an independent investigator. All participants performed the experimental tests in the mid-luteal phase of their menstrual cycle. For this standardization, ovulation was estimated by the measurement of the dates of menstruation and the duration of the menstruation for 6 months with a commercially available mobile app (Mycalendar, Period-tracker, US).

### Pre experimental trial

One week before the first trial, participants’ body weight and height were obtained by a validated scale and stadiometer (Seca 700, Hammer Steindamm, Germany) and body composition was estimated by bioimpedance (Tanita InnerScan Dual, RD-901BK36, Japan). Afterwards, participants performed a ramp exercise test to assess VO_2_max. The VO_2_max was considered valid when the following end criteria were reached at the end of the tests: VO_2_ stabilisation despite increases in ergometric power, respiratory exchange ratio greater than 1.10, and participant’s rating of perceived exertion – measured with the Borg scale from 6 to 20 points – higher than 19 points and peak heart rate superior to 80% of the age-adjusted estimate of maximum heart rate [19]. To normalize exercise intensity in the experimental trials, a regression analysis was performed for each subject for the relationship between workload (in W) and VO_2_ (L/min), as previously suggested [20]. With this association, the workloads were calculated to produce exercise intensities in the range of 30 to 80% VO_2_max. Within the week prior to the onset of the experiment, a second pre-experimental trial was carried out to familiarise participants with the protocol. In the second pre-experimental trial, participants underwent the protocol described below for the experimental trials but without the intake of any substance.

### Experimental trials

Twenty-four hours before each experimental test, participants refrained from strenuous exercise and adopted a similar diet and fluid intake regime. Participants were also required to avoid alcohol, caffeine, and other stimulants before each test. Participants were asked to complete a 24-hour dietary log the day before the first trial and to follow the same dietary pattern before the subsequent trials. In each trial, participants arrived at the laboratory (08.00–10.00 am) in a fasted state (at least 8 hours after their last meal). Upon arrival, participants voided and proper hydration prior to testing was confirmed by urine specific gravity < 1020 g/ml. Once all the standardizations were satisfied, the participants ingested the capsule assigned for the trial and rested for 60 min. In the last 5 min of the resting period, participants’ blood pressure (M6 Comfort, Omron, Japan; by triplicate) and heart rate (Wearlink+V800, Polar, Finland) were recorded. Thereafter, a urine sample was collected to assess the concentration of caffeine and paraxanthine before exercise. Then, participants performed a warm-up consisting of 10 minutes at 30% of VO_2_max and the exercise intensity was increased by 10% of VO_2max_ every 3 min until they completed the workload equivalent to 80% of VO_2_max. During exercise, expired gases were collected with a stationary breath-by-breath gas analyser (Ergostik, Geratherm Respiratory, Germany). The gas analyser used for this investigation has good inter-day reliability for respiratory measurements (CV = 7.7 ± 5.5% for the maximal rate of fat oxidation (MFO), and CV = 6.5 ± 8.0% for Fatmax) [21]. Throughout the trial, gas exchange data were averaged every 15 s. The rates of energy expenditure and substrate oxidation (fat and carbohydrate) were calculated using the non-protein respiratory quotient [22]. In each trial, the MFO was individually calculated for each participant as the highest value of fat oxidation rate obtained during the incremental exercise intensity test. The exercise intensity at which MFO was obtained for each individual was categorised as Fatmax.

After each trial, participants completed an *ad hoc* questionnaire regarding common side effects after acute caffeine intake. This questionnaire included a 1- to 10-point scale to assess the magnitude of each side effect [23]. This survey was completed on the following morning of the experiment once participants had completed their night’s sleep [24].

### Liquid chromatography–mass spectrometry (LC-MS) analysis

The assessment of urine caffeine and paraxanthine concentration was carried out through mass spectrometry coupled with an Acquity UPLC H-Class (USA). The Ultra Performance Liquid chromatography (100x2.1 mm, i.d. 1.7 μm) was performed at 30°C with an Avantor ACE (UK) C18-PFP column using a mobile phase composed of formic acid 0.1% (A) and methanol (B). Data were acquired using MRM (multiple reaction monitoring) mode. Chromatograms were integrated with MultiQuant software 1.0.3. (Sciex, Germany). Calibration curves were created using the commercial standards (caffeine VWR USA and 1.7-dimetylxanthine TCI, Japan) in the range 0.5 μg/mL-60 μg/ml.

### Statistical analysis

The results of each test were entered in a blinded fashion into the SPSS v21.0 statistical package and analysed. The data are presented as mean ± standard deviation (SD). The normality of each variable was initially tested with the Shapiro-Wilk test. A one-way analysis of variance (ANOVA) was used to compare cardiovascular variables at rest, MFO, Fatmax, urine concentrations of caffeine and paraxanthine and side-effects derived from caffeine/placebo ingestion. A two-way ANOVA (substance × exercise intensity) was used to compare energy expenditure, fat, and carbohydrate oxidation rates, heart rate and the rating of perceived exertion during exercise. Cohen’s effect size (ES) was calculated in all pairwise comparisons between caffeine doses and placebo [25]. The level of significance was set at p < 0.050.

## RESULTS

In comparison to the placebo, the acute ingestion of 3 and 6 mg/kg of caffeine did not modify resting heart rate (F_12_ = 1.130; p = 0.334), systolic blood pressure (F_12_ = 1.106; p = 0.372), diastolic blood pressure (F_12_ = 0.280; p = 0.757) or mean blood pressure (F_12_ = 0.710; p = 0.498, Table 2).

**TABLE 2:** Cardiovascular variables at rest after the acute intake of 3 and 6 mg/kg of caffeine or a placebo.

Variable (units)	Placebo	3 mg/kg	6 mg/kg	p value
Heart rate at rest (bpm)	56 ± 9	58 ± 8	57 ± 8	0.561
Systolic blood pressure (mmHg)	104 ± 12	108 ± 9	111 ± 7	0.372
Diastolic blood pressure (mmHg)	68 ± 5	70 ± 9	70 ± 7	0.757
Mean blood pressure (mmHg)	80.3 ± 5.9	82.7 ± 5.3	84.0 ± 6.3	0.498

Data are presented as mean ± standard deviations (SD).

**FIG. 1:**
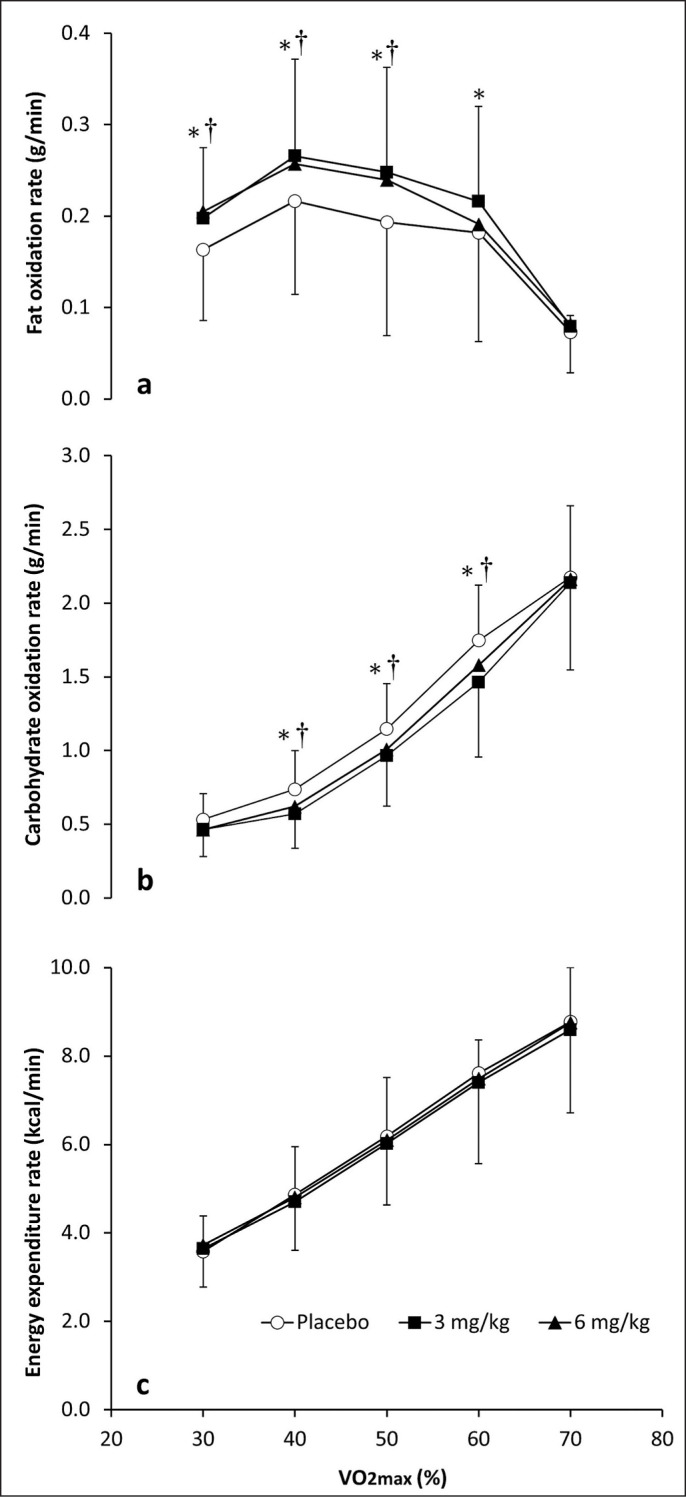
Rates of fat oxidation (a), carbohydrate oxidation (b) and energy expenditure (c) during exercise of increasing intensity after the ingestion of 3 and 6 mg/kg of caffeine or a placebo. Note: (*) Differences between placebo and 3 mg/kg (p<0.050). (†) Differences between placebo and 6 mg/kg (p<0.050).

Figure 1 depicts the effect of 3 and 6 mg/kg of caffeine on the rates of fat oxidation, carbohydrate oxidation and energy expenditure during exercise of increasing intensity. There were significant effects of substance (F_2,9_ = 5.221; p = 0.016) and exercise intensity (F_4,10_ = 31.778; p < 0.001), and a substance × exercise intensity interaction (F_8,5_ = 4.321; p = 0.025), on fat oxidation rate during exercise. The *post hoc* analysis revealed that, in comparison to the placebo, the rates of fat oxidation were higher with 3 mg/kg of caffeine at 30, 40, 50 and 60% of VO_2_max (all p < 0.050, ES from 0.42 to 0.63). Similarly, the rates of fat oxidation were higher with 6 mg/kg of caffeine than with placebo at 30, 40 and 50% of VO_2_max (all p < 0.050, ES from 0.39 to 0.58) with no differences between 3 and 6 mg/kg at any exercise intensity. There were main effects of substance (F_2,9_ = 9.632; p < 0.001) and exercise intensity (F_4,10_ = 47.522; p < 0.001), and a substance × exercise intensity interaction (F_8,5_ = 3.882; p = 0.030), on carbohydrate oxidation rate during exercise. The *post hoc* analysis revealed that the rates of carbohydrate oxidation were lower with 3 mg/kg of caffeine compared with those obtained with placebo at 40, 50 and 60% of VO_2_max (all p < 0.050, ES = 0.34 to 0.58). Similar results were found when comparing the ingestion of 6 mg/kg of caffeine vs. placebo at 40, 50 and 60% of VO_2_max (all p < 0.050, ES from 0.31 to 0.52). No differences between 3 and 6 mg/kg were observed for carbohydrate oxidation rate at any exercise intensity. There was a significant effect of exercise intensity (F_4,10_ = 46.853; p < 0.001; Figure 1c) on energy expenditure rate, whereas no effect of substance or substance × exercise intensity interaction was observed.

**FIG. 2:**
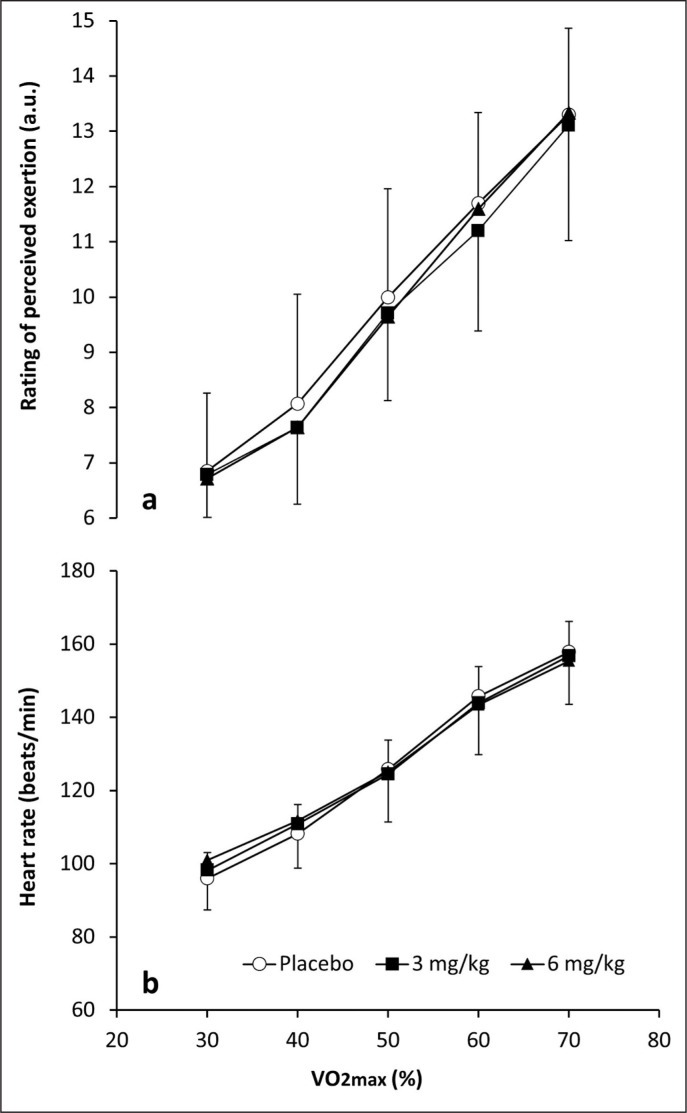
Rating of perceived exertion (a) and heart rate (b) during exercise of increasing intensity after the ingestion of 3 and 6 mg/kg of caffeine or a placebo.

Figure 2 depicts the effect both 3 and 6 mg/kg caffeine on the rating of perceived exertion and heart rate during exercise at increasing intensity. There was a significant effect of exercise intensity (F_4,10_ = 82.522; p < 0.001) on the rating of perceived exertion, while no effect of substance or substance × exercise intensity interaction was observed for this variable. There was also a significant effect of exercise intensity (F_4,10_ = 63.622; p < 0.001) on heart rate, whereas no effect of substance or substance × exercise intensity interaction was observed.

**FIG. 3:**
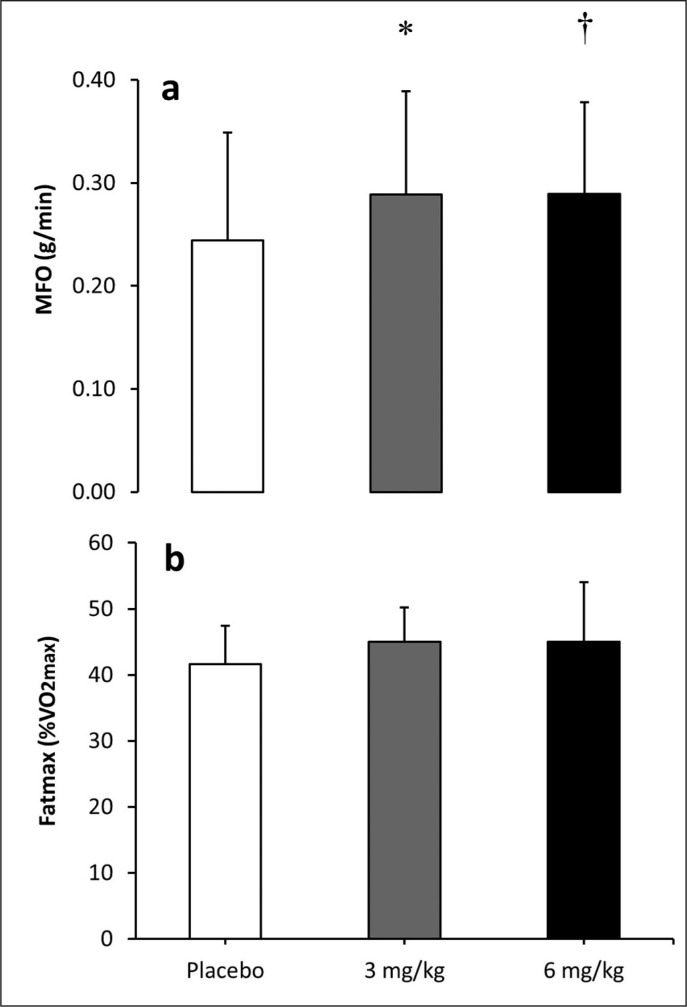
Maximal fat oxidation (MFO; (a)) and exercise intensity at maximal fat oxidation (Fatmax; (b)) during exercise of increasing intensity after the ingestion of 3 and 6 mg/kg of caffeine or a placebo. Note: (*) Differences between placebo and 3 mg/kg (p<0.050). (†) Differences between placebo and 6 mg/kg (p<0.050).

Figure 3 shows the effect of caffeine intake on MFO and Fatmax. There was an effect of substance on MFO (F_12_ = 5.311; p = 0.001). The *post hoc* analysis revealed that both 3 mg/kg (0.29 ± 0.03 g/ min, p = 0.032, ES = 0.37) and 6 mg/kg of caffeine (0.29 ± 0.04 g/ min, p = 0.042, ES = 0.34) increased MFO over the placebo condition (0.29 ± 0.03 g/min). However, the intake of caffeine did not modify the exercise intensity that elicited Fatmax (F_12_ = 0.155; p = 0.813).

Within 24 hours of caffeine ingestion, both 3 and 6 mg/kg caffeine increased the female participants’ feelings of nervousness (F_14_ = 4.215; p = 0.009) and vigour (F_14_ = 3.741; p = 0.016; Table 3), while no additional side effects were observed. There was a main effect of substance on urine caffeine concentration (F_12_ = 8.794; p = 0.001). The *post hoc* analysis showed that both 3 mg/kg (p = 0.001) and 6 mg/kg (p = 0.033) increased caffeine concentrations over the placebo condition, with no differences between doses of caffeine. There was a main effect of substance on urine caffeine paraxanthine concentration (F_12_ = 21.393; p < 0.001). The *post hoc* analysis showed that both 3 and 6 mg/kg (all p < 0.001) increased urine paraxanthine concentrations over the placebo condition, and 6 mg/kg increased it more than 3 mg/kg (p < 0.001, Table 4).

**TABLE 3:** Frequencies of adverse effects during the 24 hours following the test with the acute intake of 3 and 6 mg/kg of caffeine or a placebo.

Variable (units)	Placebo	3 mg/kg	6 mg/kg	p value
Nervousness (a.u.)	1.3 ± 0.6	3.3 ± 2.5*	3.0 ± 2.3†	**0.009**
Vigour (a.u.)	1.5 ± 0.8	3.2 ± 2.5*	3.5 ± 2.4†	**0.016**
Irritability (a.u.)	1.4 ± 0.7	1.5 ± 1.2	1.3 ± 0.5	0.732
Muscle pain (a.u.)	2.3 ± 1.9	1.5 ± 0.9	1.6 ± 1.4	0.184
Headache (a.u.)	2.5 ± 2.7	2.3 ± 2.4	1.1 ± 0.3	0.096
Gastrointestinal distress (a.u.)	1.3 ± 0.6	1.1 ± 0.4	1.9 ± 2.0	0.573
Diuresis (a.u.)	2.0 ± 1.4	2.3 ± 1.6	2.2 ± 1.7	0.821
Insomnia (a.u.)	2.6 ± 2.2	2.2 ± 1.3	2.3 ± 2.7	0.792
Sleep quality (a.u)	7.1 ± 1.7	6.5 ± 1.8	6.1 ± 2.2	0.219

Each side effect was self-reported by using 1–10 arbitrary units (a.u.) scale. Participants were previously informed that one point meant a minimal amount of that item and 10 points meant a maximal amount.

(*) Differences between placebo and 3 mg/kg (p < 0.050).

(†) Differences between placebo and 6 mg/kg (p < 0.050). Data are presented as mean ± standard deviations (SD).

**TABLE 4:** Urine caffeine and paraxanthine concentrations 45 minutes after of acute intake of 3 and 6 mg/kg of caffeine or a placebo.

Variable (units)	Placebo	3 mg/kg	6 mg/kg	p value
Urine caffeine concentration (μg/ml)	0.0 ± 0.0	3.4 ± 2.6*	3.7 ± 4.5†	**0.001**
Urine paraxanthine concentration (μg/ml)	0.0 ± 0.0	2.6 ± 0.9*	4.6 ± 3.5†^$^	**< 0.001**

(*) Differences between placebo and 3 mg/kg (p < 0.050).

(†) Differences between placebo and 6 mg/kg (p < 0.050).

($) Differences between 3 and 6 mg/kg of caffeine (p < 0.050). Data are presented as mean ± standard deviations (SD).

## DISCUSSION

In this double-blind, placebo-controlled study involving healthy active women, acute intake of caffeine of 3 and 6 mg of caffeine per kg of body mass modified substrate oxidation during an incremental protocol test on a cycle ergometer. Specifically, caffeine increased fat oxidation rates from 30 to 60% of VO_2_max and reduced carbohydrate oxidation at these workloads, independently of the caffeine dosage. The data of this study suggest that by using an appropriate exercise intensity – one that results in maximal fat oxidation during exercise – the rate of fat oxidation can be increased by 26.5% with 3 mg/kg and by 24.7% with 6 mg/kg of this substance in healthy active women.

Several recent studies using a similar incremental exercise test performed concluded that acute intake of 3 mg/kg of caffeine was effective to increase the rate of fat oxidation during exercise of low to moderate intensities (30–60% of VO_2_max) [6–8]. A recent systematic review summarizing the findings of studies published on this topic [5] concluded that caffeine was not effective to enhance fat oxidation when the dose was ≤ 3 mg/kg, while there was an increasing effect of caffeine on fat oxidation with doses of 3.1–5.9 and ≥ 6 mg/ kg of caffeine. Lastly, it was found that the effect of caffeine to enhance fat oxidation during exercise is of similar magnitude with 3 and 6 mg of caffeine per kg of body mass [11]. All these reports indicate that fat oxidation enhancement can be obtained during exercise if caffeine is ingested before exercise in the dose range of 3 to 6 mg/ kg. However, all these studies included samples of male participants or mixed samples where women formed a small part of the sample.

This current investigation is innovative because it is the first study on the effect of caffeine to enhance fat oxidation during exercise carried out with women. The study adds new information to the current evidence as it indicates that caffeine enhances fat oxidation during exercise in women while this effect is of similar magnitude independent of the dosing, at least in the 3 to 6 mg/kg range. From a simplistic viewpoint, the use of 3 mg/kg of caffeine would be more recommended than 6 mg/kg to those exercise enthusiasts seeking fat reduction during exercise because 3 mg/kg of caffeine produced an effect of similar magnitude to that observed for 6 mg/kg but with half the dose.

In the present investigation, pre-exercise resting heart rate and blood pressure were not affected by any caffeine dose. These findings are contrary to previous investigations where acute consumption of a moderate caffeine dose is associated with increments of 5–10 mmHg in diastolic blood pressure [26, 27]. However, the acute caffeine intake of the current study caused increased nervousness and vigour in the healthy active female participants (Table 3), showing similar data to those reported by a previous study in non-habituated women [28]. The low magnitude of these side effects and the low clinical relevance of these drawbacks suggest that caffeine can be considered a safe substance, at least for healthy individuals with no allergy to caffeine. However, participants should be aware of potential drawbacks of caffeine found in this and other investigations such as tolerance [29] and dependence [30], among others.

Caffeine is an alkaloid that undergoes extensive metabolism in the liver microsomes, with more than 25 metabolites identified [31]. However, the main metabolites of caffeine metabolism are paraxanthine, theobromine and theophylline [32]. In the context of sport, urine caffeine concentration has been measured to estimate the intake of caffeine [1, 33] and a threshold of 12 μg/ml was set to consider the use of caffeine as doping from 1984 to 2004. Although caffeine is no longer included in the list of banned substances, the measurement of urine caffeine concentration and its main metabolites may be useful to estimate the dose ingested. This is because the assessment of urinary caffeine and paraxanthine concentrations is an easy and reliable method without the need of any invasive protocol. In previous studies, 4.1 μg/ml of urinary caffeine in male soccer players [34] and 2.4 μg/ml in male rugby players [35] were found after the ingestion of 3 mg/kg of caffeine. In the present investigation, we observed urinary caffeine concentrations of ~3.4 and ~3.7 μg/ml for doses of 3 and 6 mg/kg of caffeine, without differences between doses. The lack of differences in urine caffeine concentration despite ingesting a 2-fold dose of caffeine in the 6 mg/kg trial may be due to the low portion of the ingested caffeine that is excreted in urine as caffeine. On the other hand, urine paraxanthine concentration was 1.8-fold higher with 6 than with 3 mg/kg of caffeine. Collectively, this information suggests that paraxanthine concentration was the urinary variable that best identified caffeine intake in healthy active female participants, as most of the caffeine orally ingested is excreted as urinary paraxanthine.

Despite the strengths and novelty of this investigation, the study has some limitations: i) It was only conducted with healthy active women, and therefore no extrapolation should be assumed for other populations such as men, elite female athletes or populations with clinical conditions. (ii) No doses lower than 3 mg/kg or higher than 6 mg/kg of caffeine were tested in this investigation. The effect of caffeine on fat oxidation may be different with higher doses of caffeine, such as 9 mg/kg, as this dose produced the greatest increase in free fatty acid concentrations in the dose-response study by Graham and Spriet [15]. However, recent literature reported that 3 and 6 mg/kg of caffeine produced an effect of similar magnitude on fat oxidation during exercise [11], suggesting the absence of a dose-response effect of caffeine on the enhancement of fat utilization as a fuel during exercise. Additionally, it is necessary to establish the minimal effective doses of caffeine for enhanced fat oxidation by investigating doses below 3 mg/kg, which has not been consistently investigated in men or women. (iii) No data on the mechanism of action of caffeine to enhance fat oxidation were obtained; a future investigation should be carried out to determine the changes in free fatty acid concentration with different doses of caffeine in women. iv) All participants were low daily caffeine consumers, which could have amplified the effect of acute caffeine intake. Future investigations should determine the effect of caffeine on fat oxidation in participants with moderate and high levels of daily caffeine intake as tolerance to caffeine’s benefits during exercise has been found with a 20-day chronic intake of the substance [29]. v) Recent evidence indicates that AA homozygotes in the -163C > A polymorphism of the cytochrome P450 1A2 gene (*CYP1A2*) may respond differently to acute caffeine intake during exercise than C-allele carriers [36], but no investigation has tested whether this polymorphism affects the individual responses to caffeine in terms of enhanced fat oxidation during exercise.

## CONCLUSIONS

In summary, acute intake of caffeine improves the utilization of fat as a fuel during aerobic exercise at 30 to 60% VO_2_max in healthy active women. The effect of caffeine on fat oxidation was of similar magnitude with 3 and 6 mg of caffeine per kg of body mass. Thus, the ingestion of at least 3 mg/kg seems to be necessary to obtain noteworthy changes in substrate oxidation during low-to-moderate intensity exercise.
